# Unraveling the Cause of Perioperative Anaphylaxis: The Role of Patent Blue Dye and Midazolam

**DOI:** 10.7759/cureus.80013

**Published:** 2025-03-04

**Authors:** Beatriz Lagarteira, Mariana Flor de Lima, Magda Bento, Cátia Santa, João Rego

**Affiliations:** 1 Anesthesiology, Unidade Local de Saúde do Tâmega e Sousa, Penafiel, PRT; 2 Allergy and Immunology, Unidade Local de Saúde do Tâmega e Sousa, Penafiel, PRT

**Keywords:** anaphylactic shock, anesthesia, midazolam, patent blue dye, perioperative

## Abstract

We report a case of anaphylactic shock in a patient scheduled for a left mastectomy with sentinel lymph node biopsy who was found to be allergic to both midazolam and patent blue dye. This case underscores the occurrence of dual drug allergy in a single patient, with the possibility of synergistic effects that cannot be ruled out. Additionally, it emphasizes the vital role of the immunoallergology team in suspected anaphylaxis cases, as their specialized knowledge is crucial for establishing an accurate diagnosis and identifying the causative agents.

## Introduction

Perioperative anaphylaxis is a life-threatening condition with an estimated mortality rate of 1.4-4.8% [1]. Notably, its fatality rate is higher than anaphylaxis in other settings, where deaths occur in less than 1% of cases [2].

During the perioperative setting, patients are exposed to numerous potential triggers of anaphylaxis, including anesthetic-related drugs, muscle relaxants, antibiotics, and intravenous fluids, as well as non-pharmacological substances such as dyes, surgical material, or latex. This concentrated exposure to multiple potential allergens within a relatively short period increases the likelihood that anesthesiologists will encounter and manage allergic reactions more frequently than other medical specialties.

According to the National Audit Project 6 (NAP6), the largest one-year prospective study on perioperative anaphylaxis, the typical signs that indicate an allergic reaction, such as rash and erythema, are observed only in 56% of cases. These symptoms may not manifest in more severe reactions until after the resuscitation phase [3]. Therefore, when a patient exhibits hypotension and tachycardia during anesthesia, a broad differential diagnosis should be considered, including severe hypovolemia, myocardial ischemia, and pulmonary embolism, with anaphylaxis always remaining a critical possibility.

## Case presentation

We present a case of a 30-year-old woman scheduled for a left total mastectomy with sentinel lymph node biopsy. The patient was previously healthy, with no surgical history or documented allergies.

Twenty minutes before induction, we performed a left serratus plane block with 20 mL of 0.375% ropivacaine. Additionally, 1 mg of midazolam and 50 mcg of fentanyl were administered for sedation during the block performance. We performed total intravenous anesthesia using a target-control infusion of remifentanil and propofol, bolus of 30 mg rocuronium with the placement of a laryngeal mask. After that, 2 g of cefazolin was also administered.

Approximately five minutes following the induction of general anesthesia and the administration of 1 mL of patent blue dye by the surgeon on the breast, the patient experienced a cardiovascular collapse. This was marked by profound hypotension, with mean arterial pressure ranging from 25 to 45 mmHg, and initial bradycardia (heart rate approximately 40 bpm). Auscultation was unremarkable, and the electrocardiogram showed no ST-segment abnormality. The hemodynamic instability exhibited minimal responsiveness to vasopressor agents, despite administering four 10 mg boluses of ephedrine and four 100 mcg boluses of phenylephrine within five minutes, resulting only in tachycardia (heart rate approximately 120 bpm). Although no visible skin rash or edema was observed at the time, the clinical presentation was rapidly interpreted as a potential anaphylactic shock.

Subsequently, we administered four boluses of 50 mcg of intravenous epinephrine and initiated a rapid infusion of 1000 mL of sodium chloride, resulting in only a partial response (mean arterial pressure <55 mmHg). Consequently, a continuous epinephrine infusion was started at 0.1 mcg/kg/min along with intravenous clemastine (2 mg) and hydrocortisone (200 mg). The hypotensive state (mean arterial pressure <60 mmHg) persisted for 30 minutes. We replaced the laryngeal mask with an orotracheal tube, and during laryngoscopy, there was no evidence of upper airway edema. She exhibited no signs of laryngospasm or bronchospasm, however, we proceeded to change the maintenance anesthesia to sevoflurane and discontinue the infusion of propofol and remifentanil. Only 20 minutes after the cardiovascular collapse, she developed a diffuse skin rash and peripheral and facial edema.

**Figure 1:**
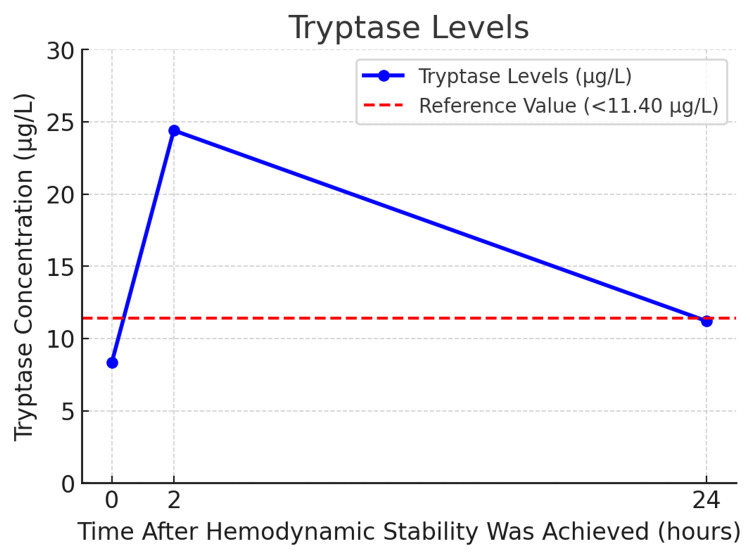
Tryptase levels at 0h, 2h and 24h after hemodynamic stability was achieved This graph depicts serum tryptase variations over three time points. The baseline level (8.33 µg/L) is below the reference threshold (11.40 µg/L). At two hours, tryptase spikes to 24.40 µg/L, indicating mast cell activation typical of anaphylaxis. By 24 hours, it declines to 11.20 µg/L, nearing baseline. This trend - an initial surge followed by a gradual decrease, reverting to normal over the next 6 to 24 hours - supports a mast cell degranulation consistent with an acute allergic reaction [4].

Due to the high suspicion of anaphylactic shock, tryptase levels were collected at moment 0 (when hemodynamic stability was achieved), 2, and 24 hours after that. The results are shown in Figure 1. Consequently, the surgical procedure was deferred, and, once her condition was stabilized, she was transferred to the Intensive Care Unit (ICU) under invasive mechanical ventilation. In the ICU, the patient was maintained on a continuous infusion of propofol (4 mg/kg/h) and boluses of rocuronium 30 mg every hour. No refractory episodes were recorded, and the patient remained hemodynamically stable throughout her stay. She was subsequently discharged to the surgical floor 24 hours after the episode.

The case was reviewed with the immunoallergology team. Given the oncological nature of the intended procedure, it was urgent to proceed with the surgery. However, continuing until the causative agent was identified was deemed unsafe. Because of the potential for false negative results, it was not feasible to conduct skin tests immediately after the incident [5].

Four weeks later, the immunoallergology team conducted skin prick tests (SPT) and intradermal tests (IDT) using a panel of agents, including midazolam, fentanyl, remifentanil, ropivacaine, cefazolin, latex, chlorhexidine, povidone iodine, and patent blue dye (Table 1). Propofol and rocuronium were not tested as they were used for anesthetic maintenance following the episode and were well tolerated. The SPT and IDT results were negative for fentanyl, remifentanil, ropivacaine, cefazolin, latex, chlorhexidine, and povidone-iodine. However, positive reactions were observed for midazolam and patent blue dye in the SPT. In the IDT, patent blue dye yielded a positive response at a 1:100 dilution and midazolam at a 1:10 dilution. These findings identified midazolam and patent blue dye as the two possible causative agents.

**Table 1:** Allergy testing results: skin prick test (SPT) and intradermal test (IDT) for perioperative agents This table shows results from allergy testing for various agents, using both the SPT and the IDT. For midazolam, the SPT is positive, and the IDT is also positive at a 1:10 dilution. For patent blue dye, both SPT and IDT are positive, with the IDT positive at a 1:100 dilution. For all other agents, both SPT and IDT results are negative.

Agent	Skin Prick Test (SPT)	Intradermal Test (IDT)
Midazolam (5mg/mL)	Positive (6x4mm)	1:100 negative; 1:10 positive
Fentanyl (0.05mg/mL)	Negative	Negative
Remifentanil (0.05mg/mL)	Negative	Negative
Ropivacaine (2mg/mL)	Negative	Negative
Cefazolin (20mg/mL)	Negative	Negative
Latex	Negative	Negative
Chlorhexidine (20mg/mL)	Negative	Negative
Patent blue dye (25mg/mL)	Positive (5x4mm)	1:100 positive
Povidone-iodine (25mg/mL)	Negative	Negative

A left mastectomy with sentinel lymph node biopsy occurred five weeks after the episode without the administration of patent blue or any benzodiazepine. During the procedure, the following agents were used: fentanyl, propofol, rocuronium, cefazolin, latex, chlorhexidine, and povidone-iodine, and it occurred without any complications. The patient was discharged one day after the surgery.

## Discussion

This case illustrates the diagnostic challenges of perioperative anaphylaxis, especially when cardiovascular collapse is the primary manifestation [6]. Here, the patient developed anaphylactic shock shortly after the induction of general anesthesia and administration of patent blue dye, presenting with severe hypotension and bradycardia followed by tachycardia that were unresponsive to vasopressors, but without respiratory symptoms such as bronchospasm or laryngospasm. Moreover, in severe cases, cutaneous manifestations like rash or angioedema are rare and usually appear only after adequate perfusion has been reestablished [3] through appropriate treatment.

The abrupt onset of cardiovascular instability following the induction of general anesthesia and administration of patent blue dye, requiring high-dose vasopressor therapy in an otherwise young and healthy patient, with no ST-segment abnormality on electrocardiogram and unremarkable findings on auscultation, heightened the suspicion of anaphylaxis while rendering alternative differential diagnoses, such as severe hypovolemia, myocardial ischemia, and pulmonary embolism, less probable.

After administring ephedrine (40 mg) and phenylephrine (400 mcg) without a response, prompt intravenous epinephrine administration and fluid resuscitation were crucial in restoring adequate arterial pressure and ensuring sufficient organ perfusion. Epinephrine works against the effects of anaphylaxis by providing vasoconstriction, bronchodilation, inotropic support, and mast cell stabilization [7]. We selected the intravenous route due to its ability to provide a rapid onset of action, as organ perfusion - including muscular and subcutaneous tissues - may be compromised during cardiovascular collapse. Furthermore, in the perioperative setting, where the patient is under continuous monitoring, the intravenous route is generally preferred, especially in cases of greater severity [8].

Notably, cutaneous symptoms only appeared approximately 20 minutes after the initial cardiovascular signs, a delayed response observed in other cases of perioperative anaphylaxis [3]. This delayed presentation highlights the variability in anaphylaxis symptoms [9], which can complicate diagnosis in the intraoperative setting, where immediate recognition is critical to patient outcomes.

Given the suspicion of anaphylaxis, serial serum tryptase measurements were obtained, as elevated tryptase levels are highly indicative of mast cell activation during allergic reactions [10]. However, while tryptase is a valuable marker, it does not identify the specific allergen, necessitating follow-up allergologic testing to pinpoint the causative agents [11,12].

Four weeks post-incident, the immunoallergology team performed comprehensive SPT and IDT on perioperatively administered agents, following established guidelines that recommend delaying testing after severe reactions to reduce false negatives [5]. Propofol and rocuronium were not tested. This decision was made by the immunoallergology team, which deemed testing unnecessary, as the patient had been exposed to these drugs for several hours after the event without any recurrence of symptoms. Both SPT and IDT identified midazolam and patent blue dye as the likely allergens, with positive reactions at a 1:10 dilution for midazolam and a 1:100 dilution for patent blue dye, consistent with known reactivity levels for these agents [13,14]. All other agents tested negative. However, as SPT and IDT have a generally low negative predictive value, negative skin test results should prompt consideration of provocation testing to confirm the absence of an allergic response [15]. Given the oncological urgency of the situation, it was imperative to proceed with surgery, which precluded the performance of provocative tests.

Five weeks after the episode, the patient underwent the planned surgery. Drugs that tested negative in SPT and IDT - specifically fentanyl, cefazolin, latex, chlorhexidine, and povidone-iodine - were used, while those with positive test results, namely patent blue dye and midazolam, were avoided. The patient’s exposure to the selected agents during this procedure effectively served as a provocation test, ruling them out as potential causes of the hypersensitivity reaction.

Based on these findings, along with the positive SPT and IDT results for midazolam and patent blue dye, these agents were identified as the most probable causative factors. Therefore, the patient received a written document for future reference in case of anesthesia or medical procedures.

Patent blue dye has been implicated in perioperative anaphylaxis, with a reported incidence of hypersensitivity reactions of 14.6 per 100.000 administrations, according to NAP6, often manifesting with cardiovascular instability [3]. Anaphylactic reactions to midazolam are uncommon but can still trigger severe hypersensitivity in rare cases [16].

This case highlights the potential for concurrent hypersensitivity to commonly used perioperative agents. Regarding the anaphylactic shock, it remains uncertain whether a single drug served as the sole trigger or if a combined allergic response contributed to the reaction.

## Conclusions

This case highlights a severe anaphylactic shock in a patient with laboratory-confirmed allergy to both midazolam and patent blue dye. It remains unclear whether one drug was the primary trigger of the reaction or if the combined allergies played a role, as a synergistic mechanism cannot be excluded. This case contributes to the literature by documenting a dual allergy to patent blue dye and midazolam in the same patient.
